# Guideline-Concordant Therapy for Community-Acquired Pneumonia in the Hospitalized Population: A Systematic Review and Meta-analysis

**DOI:** 10.1093/ofid/ofae336

**Published:** 2024-06-15

**Authors:** Chanhee Seo, Mario Corrado, Rachel Lim, Christina S Thornton

**Affiliations:** Department of Medicine, University of Calgary, Calgary, Alberta, Canada; Department of Medicine, University of Toronto, Toronto, Ontario, Canada; Department of Medicine, University of Calgary, Calgary, Alberta, Canada; Division of Respiratory Medicine, Department of Medicine, University of Calgary, Calgary, Alberta, Canada; Department of Medicine, University of Calgary, Calgary, Alberta, Canada; Division of Respiratory Medicine, Department of Medicine, University of Calgary, Calgary, Alberta, Canada; Department of Microbiology, Immunology and Infectious Diseases, University of Calgary, Calgary, Alberta, Canada

**Keywords:** antimicrobials, community-acquired pneumonia, guideline, stewardship

## Abstract

**Background:**

A commonly used guideline for community-acquired pneumonia (CAP) is the joint American Thoracic Society and Infectious Diseases Society of America practice guideline. We aimed to investigate the effect of guideline-concordant therapy in the treatment of CAP.

**Methods:**

We systematically searched MEDLINE, Embase, CENTRAL, Web of Science, and Scopus from 2007 to December 2023. We screened citations, extracted data, and assessed risk of bias in duplicate. Primary outcomes were mortality rates, intensive care unit (ICU) admission, and length of stay. Secondary outcomes were guideline adherence, readmission, clinical cure rate, and adverse complications. We performed random-effect meta-analysis to estimate the overall effect size and assessed heterogeneity using the *I^2^* statistics.

**Results:**

We included 17 observational studies and 82 240 patients, of which 10 studies were comparative and pooled in meta-analysis. Overall guideline adherence rate was 65.2%. Guideline-concordant therapy was associated with a statistically significant reduction in 30-day mortality rate (crude odds ratio [OR], 0.49 [95% confidence interval .34–.70; *I^2^* = 60%]; adjusted OR, 0.49 [.37–.65; *I^2^* = 52%]) and in-hospital mortality rate (crude OR, 0.63 [.43–.92]; *I^2^* = 61%). Due to significant heterogeneity, we could not assess the effect of guideline-concordant therapy on length of stay, ICU admission, readmission, clinical cure rate, and adverse complications.

**Conclusions:**

In hospitalized patients with CAP, guideline-concordant therapy was associated with a significant reduction in mortality rate compared with nonconcordant therapy; however, there was limited evidence to support guideline-concordant therapy for other clinical outcomes. Future studies are needed to assess the clinical efficacy and safety of current guideline recommendations.

## Teaser

In hospitalized patients with community-acquired pneumonia, guideline-concordant therapy was associated with reduced mortality rates; however, there was a lack of evidence supporting guideline-concordant therapy for improved length of stay, intensive care unit admission, readmission, clinical cure rate, and adverse complications.

Community-acquired pneumonia (CAP) is one of the leading causes of hospitalization and death affecting all age groups globally [1]. Empiric antimicrobial therapy is the mainstay of treatment of the majority of patients with pneumonia. To prevent antimicrobial misuse, resistance, and complications, an individualized risk-benefit analysis and evidence-based selection of antibiotic regimen is needed. To address this, several practice guidelines for CAP have been published [2–5].

One of the commonly referenced CAP guidelines is the American Thoracic Society (ATS) and Infectious Diseases Society of America (IDSA) guideline, first published in 2007 and updated in 2019 [3, 4]. The updated guideline reaffirms many prior recommendations including a β-lactam plus a macrolide therapy or a respiratory fluoroquinolone monotherapy for nonsevere inpatient CAP management and a β-lactam plus a macrolide or fluoroquinolone dual therapy for severe inpatient CAP management, noting subtle differences in the indications for empiric coverage for methicillin-resistant *Staphylococcus aureus* (MRSA) and *Pseudomonas* between the guidelines (Table 1).

**Table 1.** Comparison of Recommendations in the 2007 and 2019 American Thoracic Society/Infectious Diseases Society of America CAP Guidelines

Treatment Context	2007 ATS/IDSA Guideline [3]	2019 ATS/IDSA Guideline [4]
Inpatient, nonsevere or non-ICU treatment	Respiratory fluoroquinolone monotherapy β-Lactam *plus* a macrolide^a^	Respiratory fluoroquinolone monotherapy β-Lactam *plus* a macrolide^a^ If prior respiratory isolation of MRSA or *Pseudomonas*, add empiric coverage and obtain cultures to guide continued therapy If locally validated risk factors for MRSA or *Pseudomonas* present, obtain cultures but withhold empiric coverage until cultures are obtained
Inpatient, severe or ICU treatment	β-Lactam *plus* azithromycin or fluoroquinolone For *Pseudomonas* infection, an antipneumococcal, antipseudomonal β-lactam *plus* ciprofloxacin or levofloxacin or aminoglycoside + azithromycin or aminoglycoside + antipneumococcal fluoroquinolone For MRSA infection, add vancomycin or linezolid	A β-Lactam *plus* macrolide or fluoroquinolone^b^ If prior respiratory isolation of MRSA or *Pseudomonas*, add empiric coverage and obtain cultures to guide continued therapy If locally validated risk factors for MRSA or *Pseudomonas* present, add empiric coverage and obtain cultures to guide continued therapy

Abbreviations: ATS, American Thoracic Society; ICU, intensive care unit; IDSA, Infectious Diseases Society of America; MRSA, methicillin-resistant *Staphylococcus aureus.*

^a^Doxycycline can be used as an alternative to the macrolide.

^b^Doxycycline is an alternative to the macrolide and fluoroquinolone.

Several studies have associated the use of guideline-concordant CAP therapy with improved mortality rates among patients with pneumonia [6–9]. However, criticism has been raised about potential overtreatment and misuse of empiric broad-spectrum therapy based on MRSA and *Pseudomonas* risk factors alone, with discordance in the recommended therapy and local epidemiology and antimicrobial resistance patterns in different areas of the world [10]. To date, there has been no comprehensive review of the clinical outcomes following ATS/IDSA guideline–concordant CAP therapy to our knowledge. Therefore, the current study aimed to systematically review the literature and meta-analyze all available studies to provide an evidence-based appraisal of the following clinical questions pertaining to the ATS/IDSA guideline–concordant care for adults hospitalized with CAP: (1) What are the outcomes of CAP treated with ATS/IDSA guideline–concordant therapy, including mortality rate, length of stay (LOS), and intensive care unit (ICU) admission? (2) What is the prevalence of ATS/IDSA guideline–concordant CAP treatment? And (3) What are the readmission and clinical cure rates and adverse complications with guideline-concordant versus nonconcordant therapy?

## METHODS

### Protocol Registration

This systematic review was registered in the Open Science Framework Registries (https://doi.org/10.17605/OSF.IO/X8VFA) and followed the statement of Preferred Reporting Items for Systematic Reviews and Meta-analysis (PRISMA) reporting guidelines (Supplementary Figure 1) [11].

### Search Strategy

We performed a systematic search in MEDLINE (Ovid), Embase (Ovid), Cochrane Central Register of Controlled Trials (CENTRAL), Web of Science, and Scopus using a predefined search strategy that consisted of a Boolean combination of medical subject headings and keywords to identify studies on guideline-concordant therapies for CAP (filtered by English language and adult population, defined as age ≥18 years) (Supplementary Figure 2). We manually screened the bibliographies of relevant articles to identify potentially eligible studies that were not captured in the initial database search. We limited the search results to articles indexed from 2007 (ie, when the first joint CAP ATS/IDSA guideline was published) through 9 December 2023.

### Study Eligibility Criteria

Included studies were (1) quantitative or epidemiological peer-reviewed primary articles; (2) cross-sectional, observational cohort, longitudinal, case-controlled, quasi-experimental, and randomized controlled trials; (3) on adults (ie, ≥ 18 years of age) with diagnosed CAP requiring hospitalization; (4) concordant with the ATS/IDSA 2007 or 2019 CAP guidelines for the study intervention, as determined by study authors (ie, use of guideline recommended antimicrobials according to disease severity and risk factors); and (5) reporting on outcome measures of interest as listed below. We excluded studies that were (1) primarily focused on the pediatric population, (2) on hospital-acquired or ventilator-associated pneumonia, or (3) commentaries, case studies, reviews, editorial, letters to the editor, or opinion articles.

### Study Selection

We used COVIDence (Veritas Health Information) as the primary screening tool. Citations were imported from each database and duplicates were removed. Two reviewers (C. S. and M. C.) independently screened the included articles in duplicate, and any discrepancies were resolved by consensus. A 2-step screening process was conducted. First, we performed a title-abstract screening based on the exclusion criteria and removed all articles that were deemed to be irrelevant for this review. Then we performed a full-text screening of the remaining articles and included articles that satisfied all the inclusion criteria. In both steps, we performed a calibration on the first 20 articles to ensure satisfactory interrater reliability (>80%).

### Data Extraction

We used a standardized data collection table to extract relevant data on study characteristics (ie, study identifier, design, time frame, country, and guideline edition), patient population (ie, population size, age, and sex), CAP outcome measures including adjusted variables for overall effect estimates when available. Two reviewers (C. S. and M. C.) independently extracted data in duplicate, and any discrepancies were resolved by consensus. This study did not include factors necessitating patient consent.

### Outcomes

Primary outcomes of this study included the mortality rate (ie, in-hospital and 30-day rates), hospital LOS, and ICU admission rate. Secondary outcomes included clinical cure rate, readmission rate, any complications related to guideline-concordant and nonconcordant therapies, and overall adherence rate of guideline-concordant therapy in treating CAP.

### Risk of Bias Assessment and Certainty of Evidence Assessment

We assessed the risk of bias of individual studies using the Risk Of Bias In Nonrandomized Studies-of Exposure (ROBINS-E) tool [12]. Two reviewers (C. S. and M. C.) independently appraised the risk of bias of included studies and any discrepancies were resolved by consensus. We assessed the overall evidence level of outcomes using the Grading of Recommendations, Assessment, Development and Evaluation (GRADE) tool [13]. The overall evidence was summarized as very low, low, moderate, or high.

### Statistical Analysis

Due to anticipated heterogeneity between studies, we performed meta-analyses using a random-effect model when ≥2 studies reported on the same outcome measures to calculate a pooled odds ratio (OR) and 95% confidence interval (CI) values. We pooled the data by computing ORs when the number of events were reported; otherwise, we pooled the logarithm of the ORs using an inverse variance approach. We performed separate meta-analyses for studies that reported adjusted ORs (aORs) as opposed to crude ORs. For continuous variables (ie, LOS), we converted individual effect sizes into standardized mean differences and corresponding 95% CIs to meta-analyze. We planned to perform subgroup analyses of the primary outcomes according to the different ATS/IDSA guideline editions, disease severity (eg, CURB-65 and the pneumonia severity index), and general ward versus ICU destination. However, due to a lack of studies reporting outcomes using the 2019 ATS/IDSA guideline and disease severity, only a subgroup analysis of ward versus ICU destination was performed.

We assessed the heterogeneity among included studies using *I*^2^ statistics with the thresholds of 25%, 50%, and 75% representing low, medium, and high heterogeneity respectively [14]. When there was considerable heterogeneity (ie, *I*^2^ ≥ 50%), we performed a sensitivity analysis by comparing study-level characteristics and repeating the analysis using a subset of the data that minimized variations between the included studies (ie, high-quality vs low-quality studies, prospective cohort vs retrospective cohort, and leave-one-out analysis). If there remained persistently significant heterogeneity, we performed narrative synthesis instead to report the outcomes.

To assess publication bias, we first visually examined funnel plots, and if >10 studies were included in the analysis, we used the Egger test of regression and Begg and Mazumdar's rank correlation test to determine the significance of funnel plot asymmetry [15–17]. We considered differences to be statistically significant at *P* ≤ .05. All statistical analyses and meta-analyses were performed using the Cochrane Collaboration's RevMan Web and R software (version 4.3.2).

## RESULTS

### Search Results

**Figure 1.**
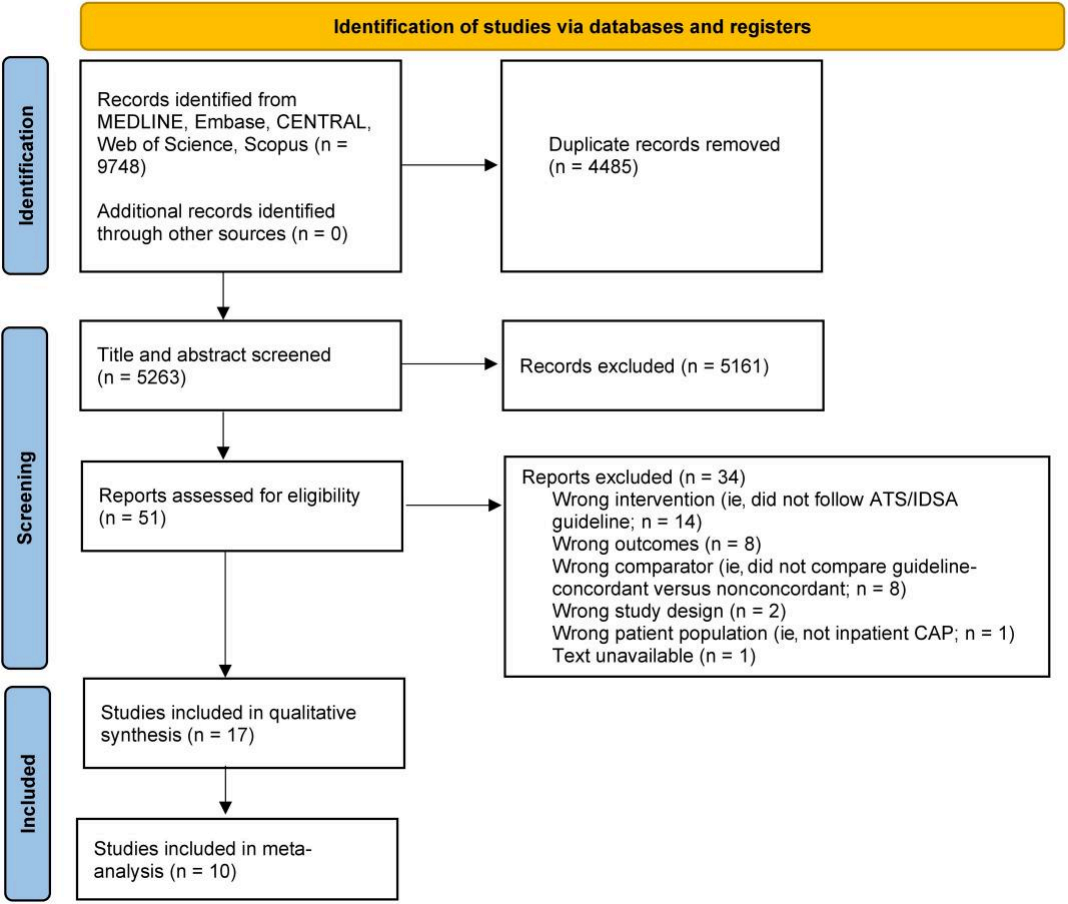
PRISMA flow diagram of the study screening process. Abbreviations: ATS, American Thoracic Society; CAP, community-acquired pneumonia; IDSA, Infectious Diseases Society of America.

The electronic search of the aforementioned databases retrieved 9748 citations, of which 5263 remained after duplicates were removed (Figure 1). After screening for title-abstract, a total of 5212 articles were excluded. No additional articles were identified from the manual bibliography screen. The full texts of the remaining 51 articles were assessed for inclusion. In total, 17 articles met the inclusion criteria for this review [6–9, 18–30], of which 10 were comparative and fulfilled criteria for meta-analysis [6–8, 20, 21, 24, 25, 27, 29, 30]. There was high interrater reliability (κ > 95%) in all steps of the screening process.

### Study Characteristics

The characteristics of the included studies are collated (Table 2). Studies were universally observational in nature (15 retrospective cohort, 1 prospective cohort, and 1 case-control study), with a total patient population of 82 240. Fourteen studies followed the 2007 and 3 the 2019 ATS/IDSA guideline. Of these, 5 studies assessed the impact of guideline-concordant therapy in an ICU setting. Only 1 study assessed the clinical impact of guideline-concordant therapy in patient populations at high risk for MRSA [23]. Studies were conducted mainly in the United States (n = 5), Canada (n = 2), Japan (n = 2), South Korea (n = 1), Taiwan (n = 1), New Zealand (n = 1), Saudi Arabia (n = 1), and Spain (n = 1). Three studies were conducted in Europe.

**Table 2.** Study Characteristics of Included Primary Studies

Authors	Year of Publication	Country	Study Design	Study Period	ATS/IDSA Guideline Version	Sample Size, No. of Patients	Setting	Adherence to Guideline, No. of Patients/Total (%)
Aikman et al [18]	2013	New Zealand	RC	2007	2007	177	NS	93/177 (53)
Alessa et al [19]	2023	Saudi Arabia	RC	2019–2021	2019	124	NS	68/124 (55)
Arnold et al [20]	2009	Multiple	RC	2001–2007	2007	1649	NS	975/1649 (59)
Cilloniz et al [21]	2015	Spain	RC	2000–2013	2007	643	NS	437/643 (68)
Frei et al [7]	2010	USA	RC	1999–2000	2007	129	ICU	53/129 (41)
Grenier et al [8]	2011	Canada	RC	1997–2008	2007	391	NS	268/391 (69)
Ishiguro et al [22]	2013	Japan	RC	2002–2011	2007	1032	NS	687/1032 (67)
Johnson et al [9]	2014	USA	RC	2001–2007	2007	1946	ICU	1519/1946 (78)
Jones et al [23]	2020	USA	RC	2008–2013	2019	19 045	NS	12 714/19 045 (67)
Kang et al [24]	2021	South Korea	RC	2013–2019	2007	630	NS	359/630 (60)
Kobayashi et al [25]	2022	Japan	RC	2013–2014	2019	721	NS	257/416 (62)
Lee et al [26]	2013	Taiwan	RC	2007–2008	2007	208	NS	148/208 (71)
Martin-Loeches et al [27]	2010	Multiple	PC	NR	2007	218	ICU	100/218 (46)
McCabe et al [6]	2009	USA	RC	1999–2003	2007	54 619	GW	35 477/54 619 (65)
Pflanzner et al [28]	2019	Canada	RC	2016–2017	2007	87	ICU	48/87 (55)
Rello et al [29]	2017	Multiple	Case-control	2000–2002; 2008–2015	2007	333	ICU	192/333 (58)
Sims et al [30]	2012	USA	RC	2009	2007	288	NS	197/288 (68)

Abbreviations: ATS, American Thoracic Society; GW, general ward; ICU, intensive care unit; IDSA, Infectious Diseases Society of America; NR, not reported; NS, not specified; PC, prospective cohort; RC, retrospective cohort.

### Risk of Bias and Publication Bias

The risk of bias assessment for the included studies is reported in Supplementary Figure 3. Overall, 5 studies were deemed low risk, 10 studies were deemed to have some concerns for risk of bias, and 2 studies were deemed to have high risk of bias. We did not detect strong evidence of publication bias based on funnel plot symmetry (Supplementary Figure 4). Because there were <10 individual studies in each meta-analysis, we could not perform the Egger test of regression or Begg and Mazumdar's rank correlation test to quantitatively assess publication bias.

### Mortality Rates

**Figure 2.**
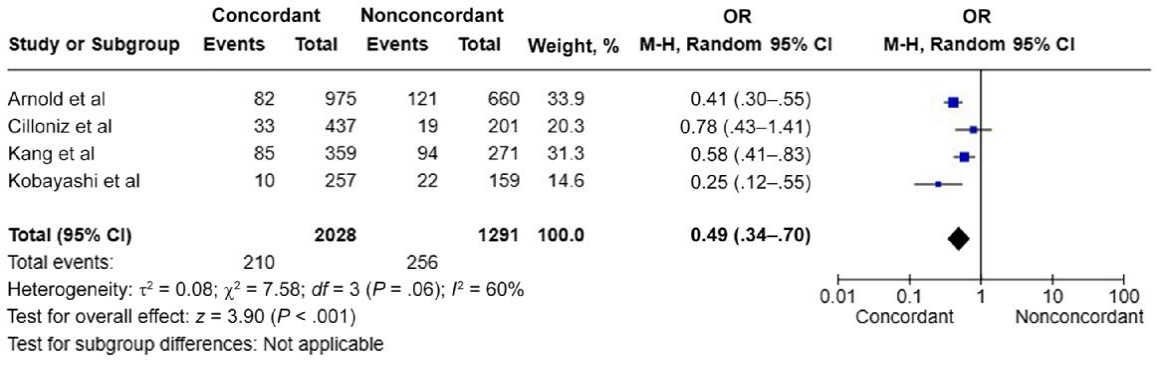
Meta-analysis comparing unadjusted 30-day mortality outcomes between guideline-concordant and nonconcordant therapy [20, 21, 24, 25]. Abbreviations: CI, confidence interval; M-H, Mantel-Haenszel; OR, odds ratio.

Across unadjusted studies performed in a non-ICU setting, a strong association was found between guideline-concordant therapy and reduced 30-day mortality rate (k = 4; n = 3319; OR, 0.49 [95% CI, .34–.70]; *I^2^* = 60%) (Figure 2) [20, 21, 24, 25]. A similar 30-day mortality trend was found across adjusted studies performed in a non-ICU setting (*k* = 2 [number of primary studies]; n = 1112; aOR, 0.49 [95% CI, .37–.65]; *I^2^* = 52%) (Supplementary Figure 5) [8, 25].

**Figure 3.**
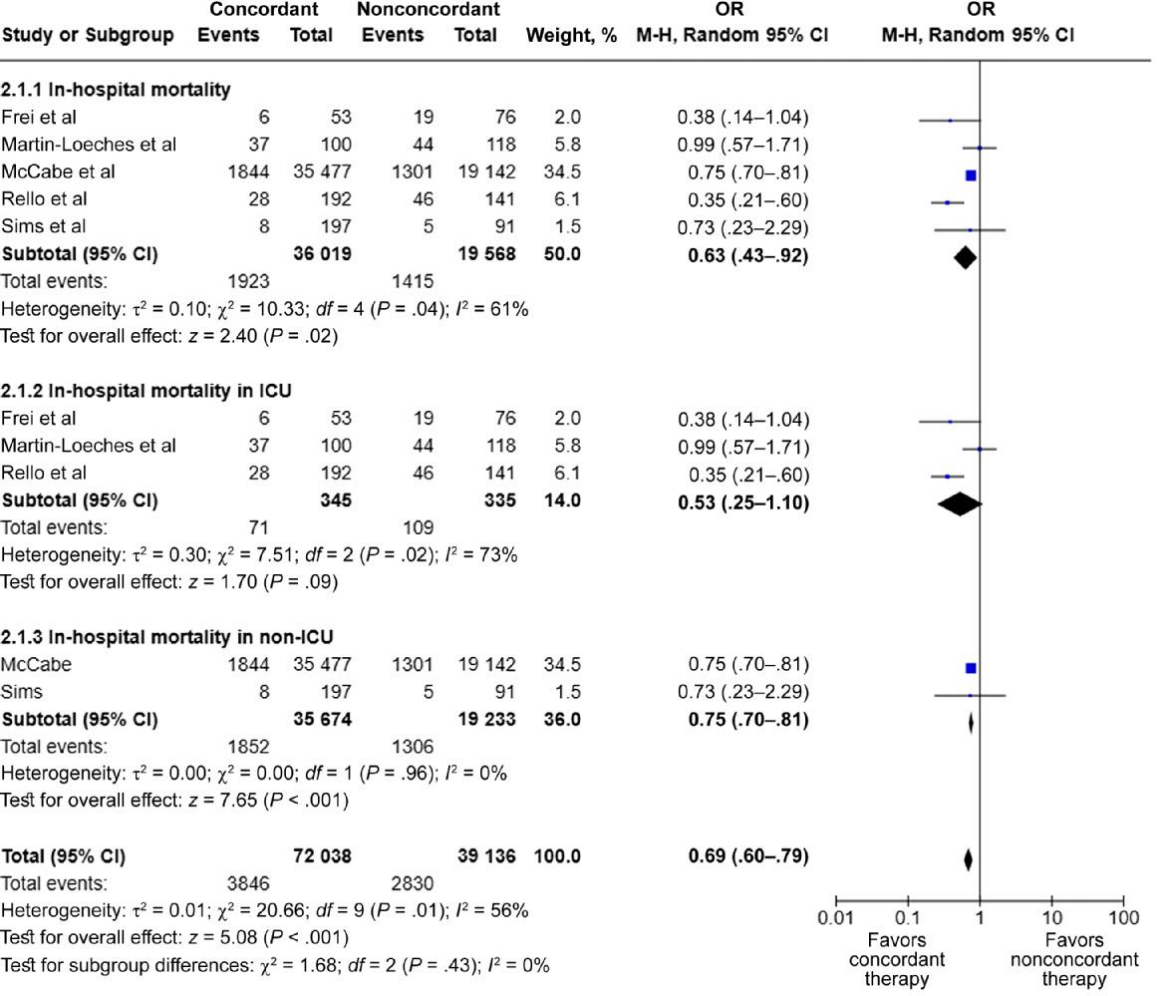
Meta-analysis of unadjusted in-hospital mortality outcomes between guideline-concordant and nonconcordant therapy [6, 7, 27, 29, 30]. Abbreviations: CI, confidence interval; ICU, intensive care unit; M-H, Mantel-Haenszel; OR, odds ratio.

Across unadjusted studies performed in both non-ICU and ICU settings, a strong association was found of guideline-concordant therapy with reduced in-hospital mortality rate (*k* = 5; n = 55 587; OR, 0.63 [95% CI, .43­–.92]; *I^2^* = 61%) (Figure 3) [6, 7, 27, 29, 30]. This trend persisted when the analysis was narrowed to a non-ICU setting only (*k* = 2; n = 54 907; OR, 0.75 [95% CI, .70–.81]; *I^2^* = 0%) [6, 30] but not in an ICU setting (*k* = 3; n = 680; OR, 0.53 [.25–1.10]; *I^2^* = 73%) (Figure 3) [7, 27, 29]. However, when aORs were pooled, a strong association persisted between guideline-concordant therapy and reduced in-hospital mortality rate in an ICU setting (*k* = 2; n = 462; aOR, 0.59 [95% CI, .44–.80]; *I^2^* = 0%) (Supplementary Figure 5) [7, 29].

Among those deemed at high risk for MRSA pneumonia, one study reported that patients who appropriately received guideline-concordant anti-MRSA therapy due to ICU admission (adjusted risk ratio [aRR], 1.3 [95% CI, 1.2–1.5]), high clinical risk for MRSA (aRR, 1.2 [1.1–1.4]), or positive MRSA surveillance screen (aRR, 1.6 [1.3–1.9]) actually had a higher 30-day mortality risk than those who did not receive empiric MRSA coverage [23].

In a sensitivity analysis excluding low-quality studies, there was persistently significant association between guideline-concordant therapy and reduced 30-day and in-hospital mortality rates (Supplementary Figure 6). A similarly strong association was found in a leave-one-out analysis for 30-day mortality rate. However, when the study by McCabe et al [6] was excluded in a leave-one-out analysis for in-hospital mortality rate, there was no longer a significant association, although the general trend remained the same (OR, 0.56 [95% CI, .31–1.02]; *P* = .06; *I^2^* = 61%).

### ICU Admission

Three studies reported outcomes on ICU admission when patients were treated with guideline-concordant versus nonconcordant therapy. Individually, Grenier et al [8] and Sims et al [30] reported statistically significant reductions in ICU admission when patients were treated with guideline-concordant therapy (OR, 0.17 [95% CI, .10–.29] and 0.45 [.27–.75], respectively) while Cilloniz et al [21] found no significant association (1.42 [.96–2.08]). When the outcomes from the 3 studies were combined, there was no significant association between the receipt of guideline-concordant therapy and ICU admission, barring significant heterogeneity between the studies (*I*^2^ = 95%) (Supplementary Figure 6). Insignificant effect size and high heterogeneity persisted even after leave-one-out sensitivity analysis was performed (Supplementary Figure 7).

### Hospital LOS

Four studies reported outcomes on hospital LOS when patients were treated with guideline-concordant versus nonconcordant therapy. Individually, Arnold et al [20] found that the median LOS (interquartile range) for those who received guideline-concordant therapy (8 [5–15] days) was significantly shorter than for those who did not (10 [10–14] days]; *P* = .01). A similar trend was reported by Sims et al [30] (mean, 5.0 ± 5.0 days for concordant vs 7.0 ± 5.1 days for nonconcordant therapy; *P* = .002). In contrast, Frei et al [7] reported no significant association between the receipt of guideline-concordant therapy and LOS (aOR, 0.87 [95% CI, .57–1.31]), and Cilloniz et al [21] reported that those who received nonconcordant therapy actually had a shorter LOS than those who received concordant therapy (median LOS [interquartile range], 6 [4–12] vs 8 [5–13] days, respectively; *P* = .001). Meta-analysis of the LOS outcomes could not be performed due to varying outcome measures reported by the studies.

### Secondary Outcomes

Only 1 study compared the clinical cure rate between guideline-concordant therapy and nonconcordant therapy. Ishiguro et al [22] found that patients who received guideline-concordant therapy were more likely to be clinically cured when compared with those who received nonconcordant therapy (612 of 687 [89.1%] vs 290 of 345 [84.1%]; *P* = .03). Similarly, only 1 study compared the readmission rates between guideline-concordant and nonconcordant therapy. Sims et al [30] reported an insignificant difference in 30-day readmission rates between those who received guideline-concordant and those who received nonconcordant therapy (38 of 197 [19.3%] vs 20 of 91 [22.0%], respectively; *P* = .60).

Three studies reported adverse complications experienced by patients receiving guideline-concordant therapy versus nonconcordant therapy. Cilloniz et al [21] reported increased risk of developing acute renal failure among patients treated with guideline-concordant therapy (36% vs 21%; *P* < .001) but McCabe et al [6] reported the opposite (aOR, 0.79 [95% CI, .67–.94]). Similar contrasting data were reported for respiratory failure/acute respiratory distress syndrome, wherein Cilloniz et al reported increased risk following guideline-concordant therapy (7% vs 3%; *P* = .03) while McCabe et al reported no significant association (aOR, 0.97 [95% CI, .85–1.10]), and for sepsis/shock, wherein Cilloniz et al reported no significant association (12% vs 12%; *P* = .78) while McCabe et al reported reduced risk following guideline-concordant therapy (aOR, 0.83 [95% CI, .72–.96]). Finally, Ishiguro et al [22] found that, compared with discordant therapy, guideline-concordant therapy resulted in fewer polymicrobial infections (10.9% vs 15.9%; *P* = .03).

The adherence rate for guideline-concordant CAP therapy across the 17 included studies was 65.2% overall, 65.0% for the 2007 guideline, and 65.5% for the 2019 guideline (Table 2). When grouped by ICU versus non-ICU setting, 70.5% of patients who were admitted to the ICU received guideline-concordant therapy, compared with 65.0% in non-ICU patients. When grouped by continent, North America had the highest adherence rate (*k* = 7 [65.7%]), followed by Europe (*k* = 3 [61.1%]), Asia (*k* = 4 [56.0%]), Middle East (*k* = 1 [54.8%]), and Oceania (*k* = 1 [52.5%]).

### Certainty of Evidence Assessment

There were serious or very serious concerns with 2 GRADE domains (risk of bias [ROB], inconsistency) across the 4 studies that reported 30-day mortality rates (Supplementary Figure 8). The overall certainty of the evidence was very low for guideline-concordant therapy on reducing 30-day mortality rates among hospitalized patients with CAP. There were further serious or very serious concerns with 3 GRADE domains (ROB, inconsistency, imprecision) across the 5 studies that reported in-hospital mortality rates. The overall certainty of evidence was similarly very low for guideline-concordant therapy on reducing in-hospital mortality rates among hospitalized patients with CAP. Finally, there were serious or very serious concerns with 4 GRADE domains (ROB, inconsistency, indirectness, imprecision) across 3 and 4 studies that reported ICU admission and hospital LOS, respectively. The overall certainty of evidence was very low for both outcomes.

## DISCUSSION

In this systematic review of 17 observational studies and meta-analysis of 10 comparative studies enrolling 59 621 patients hospitalized for CAP, we found a statistically significant reduction in 30-day mortality rate for non-ICU patients and in-hospital mortality rate for both non-ICU and ICU patients when they were treated with ATS/IDSA guideline–concordant therapy compared with nonconcordant therapy. However, there were serious concerns with ≥2 GRADE domains, resulting in a very low certainty of evidence for guideline-concordant therapy for all 4 primary outcomes. Moreover, due to significant between-study heterogeneity and limited available evidence, no definitive comparative conclusion could be made for hospital LOS, ICU admission, readmission and clinical cure rates, and adverse complications between guideline-concordant and nonconcordant therapies.

Although the pooled OR is suggestive of the overall mortality benefit of guideline-concordant therapy, administration of empirical anti-MRSA therapy in high-risk patients as recommended by the ATS/IDSA CAP guidelines was counterintuitively associated with an increased 30-day mortality risk [23]. The observation that guideline-concordant broad-spectrum antibiotic treatment is associated with higher mortality rates and adverse complications has been also reported in other patient populations, such as those with healthcare-associated pneumonia and community-onset sepsis [31–33]. Notably, since publication of the ATS/IDSA pneumonia guidelines [3, 4] and the Surviving Sepsis Campaign guidelines [34], the use of broad-spectrum antibiotics (ie, vancomycin and piperacillin-tazobactam) has increased, yet the rates of isolation of multidrug-resistant pathogens, including MRSA and *Pseudomonas*, and overall clinical outcomes of pneumonia have remained unchanged [25, 35, 36].

In this context, the clinical validity of the ATS/IDSA CAP guidelines’ recommendation of initiating empirical broad-spectrum antibiotics in patients at high risk for MRSA and *Pseudomonas* is elusive. Previous studies have shown that pathogen-directed antibiotic treatment in patients with CAP is feasible and noninferior to empirical broad-spectrum antibiotics [37, 38]. Further research is needed to assess the clinical benefits and harms of empirical broad-spectrum antibiotics in high-risk patient populations to identify effective antibiotic decision-making strategies for patients admitted with CAP and subsequent postempirical treatment de-escalation.

Our findings did not identify convincing evidence supporting guideline-concordant therapy for other clinical measures, including hospital LOS, ICU admission, readmission and clinical cure rates, and adverse complications. While this highlights a paucity in available data pertaining to clinical outcomes following guideline-concordant therapy, we also noted considerable heterogeneity between the few studies that did report such outcomes. One possible explanation of the observed heterogeneity between the studies may be in part due to different local prevalence of bacterial pathogens responsible for CAP. For instance, Peto et al [39] reported in their systematic review of 48 studies that *Streptococcus pneumoniae* was found in much higher numbers of hospitalized patients with CAP in Europe (25.9%) compared with Asia (13.3%). Conversely, gram-negative enteric bacteria and *S aureus* were more commonly implicated in hospitalized CAP cases in Asia (9.0% and 4.0%, respectively) than in Europe (2.7% and 1.4%, respectively).

Considering the implications of bacterial etiology in pneumonia severity and mortality rates [40–42], such differing trends in local prevalence pattern are important. Moreover, it has been repeatedly reported that different geographic locations harbor different antimicrobial resistance patterns for pathogens responsible for CAP [43–45]. These findings call into question whether recommendations outlined in the ATS/IDSA CAP guidelines are generalizable to other areas of the world and add urgency to the need for further research into the clinical utility and relevance of the CAP guidelines across different patient populations to determine the best treatment options for this very common disease.

We acknowledge several limitations to this systematic review and meta-analysis. First, all included studies were observational and variably adjusted for covariates, which may in turn suffer from selection, confounding, and recall bias. However, while limited to mortality outcomes only, both unadjusted and adjusted random-effect meta-analyses supported the main findings with consistently large effect estimates. Although we were cautious not to take the computed effect estimates at face value, especially considering the nonnegligible heterogeneity in the meta-analyses, the overall qualitative effect and the direction of association between guideline-concordant therapy and mortality benefit in CAP management are still likely of high certainty. Second, as previously described, the pooled effect estimates in this study demonstrated considerable between-study heterogeneity, although this variability was diminished between adjusted studies. While the *I*^2^ statistics are often inflated in meta-analyses of observational studies [46], the findings presented in this study should be interpreted with caution. Future studies should strive to adjust for potential confounders in analysis when reporting outcomes.

Third, the pooled effect size for in-hospital mortality rate showed inconsistent result in a leave-one-out sensitivity analysis when McCabe et al [6] was excluded. While this was likely due to the considerable heterogeneity between remaining studies as above, McCabe et al also relied on *International Classification of Diseases* codes to identify patient data, which could lead to considerable false-positives [47–49]. Considering that McCabe et al had the largest study included in our meta-analysis, this inconsistency in the sensitivity analysis further weakens the overall validity of our analysis. Fourth, nearly all studies evaluated did not specify what constituted guideline concordant and nonconcordant therapies, limiting data analysis. Finally, while 3 studies [19, 23, 25] included in this review followed the 2019 ATS/IDSA guideline, only one provided comparative data that was pooled in meta-analysis [25]. Although there is considerable overlap between the 2007 and 2019 CAP guidelines, future studies investigating the clinical impact of the recommendations outlined in the most recent guideline are urgently needed to assess their efficacy and validity.

In summary, these findings suggest a mortality benefit of guideline-concordant therapy per the ATS/IDSA CAP guidelines; however, the strength of this association is attenuated by between-study heterogeneity and overall very low certainty of evidence per the GRADE evidence synthesis. Moreover, we were not able to discern conclusions regarding the superiority of guideline-concordant therapy for hospital LOS, ICU admission, readmission and clinical cure rates, and adverse complications. Further robust studies are needed to definitively conclude the clinical efficacy and safety of guideline-concordant therapy as recommended by the ATS/IDSA guidelines.

## Supplementary Material

ofae336_Supplementary_Data

## Supplementary Data

Supplementary materials are available at *Open Forum Infectious Diseases* online. Consisting of data provided by the authors to benefit the reader, the posted materials are not copyedited and are the sole responsibility of the authors, so questions or comments should be addressed to the corresponding author.

## References

[1] Troeger C, Forouzanfar M, Rao PC, et al Estimates of the global, regional, and national morbidity, mortality, and aetiologies of lower respiratory tract infections in 195 countries: a systematic analysis for the global burden of disease study 2015. Lancet Infect Dis 2017; 17:1133–61. 28843578 10.1016/S1473-3099(17)30396-1 PMC5666185

[2] Woodhead M, Blasi F, Ewig S, et al Guidelines for the management of adult lower respiratory tract infections—full version. Clin Microbiol Infect 2011; 17(suppl 6):E1–E59. 10.1111/j.1469-0691.2011.03672.x PMC7128977 21951385

[3] Mandell LA, Wunderink RG, Anzueto A, et al Infectious Diseases Society of America/American Thoracic Society consensus guidelines on the management of community-acquired pneumonia in adults. Clin Infect Dis 2007; 44(suppl 2):S27–72. 17278083 10.1086/511159 PMC7107997

[4] Metlay JP, Waterer GW, Long AC, et al Diagnosis and treatment of adults with community-acquired pneumonia: an official clinical practice guideline of the American Thoracic Society and Infectious Diseases Society of America. Am J Respir Crit Care Med 2019; 200:e45–67. 31573350 10.1164/rccm.201908-1581ST PMC6812437

[5] Lim WS, Baudouin SV, George RC, et al BTS guidelines for the management of community acquired pneumonia in adults: update 2009. Thorax 2009; 64(suppl 3):iii1–iii55. 19783532 10.1136/thx.2009.121434

[6] McCabe C, Kirchner C, Zhang H, Daley J, Fisman DN. Guideline-concordant therapy and reduced mortality and length of stay in adults with community-acquired pneumonia: playing by the rules. Arch Intern Med 2009; 169:1525. 19752411 10.1001/archinternmed.2009.259

[7] Frei CR, Attridge RT, Mortensen EM, et al Guideline-concordant antibiotic use and survival among patients with community-acquired pneumonia admitted to the intensive care unit. Clin Ther 2010; 32:293–9. 20206787 10.1016/j.clinthera.2010.02.006

[8] Grenier C, Pepin J, Nault V, et al Impact of guideline-consistent therapy on outcome of patients with healthcare-associated and community-acquired pneumonia. J Antimicrob Chemother 2011; 66:1617–24. 21586592 10.1093/jac/dkr176

[9] Johnson CS, Frei CR, Metersky ML, Anzueto AR, Mortensen EM. Non-invasive mechanical ventilation and mortality in elderly immunocompromised patients hospitalized with pneumonia: a retrospective cohort study. BMC Pulm Med 2014; 14:7. 24468062 10.1186/1471-2466-14-7 PMC3914374

[10] Pletz MW, Blasi F, Chalmers JD, et al International perspective on the new 2019 American Thoracic Society/Infectious Diseases Society of America community-acquired pneumonia guideline: a critical appraisal by a global expert panel. Chest 2020; 158:1912–8. 32858009 10.1016/j.chest.2020.07.089 PMC7445464

[11] Shamseer L, Moher D, Clarke M, et al Preferred Reporting Items for Systematic Review and Meta-analysis Protocols (PRISMA-P) 2015: elaboration and explanation. BMJ 2015; 349:g7647–g7647. 10.1136/bmj.g7647 25555855

[12] Higgins J, Morgan R, Rooney AA, et al; ROBINS-E Development Group. Risk of Bias in Non-randomized Studies - of Exposure (ROBINS-E). Published 20 June 2023. Available at: https://www.riskofbias.info/welcome/robins-e-tool. Accessed 21 February 2024.

[13] Guyatt GH, Oxman AD, Vist GE, et al GRADE: an emerging consensus on rating quality of evidence and strength of recommendations. BMJ 2008; 336:924–6. 18436948 10.1136/bmj.39489.470347.AD PMC2335261

[14] Higgins JPT, Thompson SG, Deeks JJ, Altman DG. Measuring inconsistency in meta-analyses. BMJ 2003; 327:557–60. 12958120 10.1136/bmj.327.7414.557 PMC192859

[15] Egger M, Smith GD, Schneider M, Minder C. Bias in meta-analysis detected by a simple, graphical test. BMJ 1997; 315:629–34. 9310563 10.1136/bmj.315.7109.629 PMC2127453

[16] Begg CB, Mazumdar M. Operating characteristics of a rank correlation test for publication bias. Biometrics 1994; 50:1088–101. 7786990

[17] Lau J, Ioannidis JPA, Terrin N, Schmid CH, Olkin I. The case of the misleading funnel plot. BMJ 2006; 333:597–600. 16974018 10.1136/bmj.333.7568.597 PMC1570006

[18] Aikman KL, Hobbs MR, Ticehurst R, Karmakar GC, Wilsher ML, Thomas MG. Adherence to guidelines for treating community-acquired pneumonia at a New Zealand hospital. J Pharm Pract Res 2013; 43:272–5.

[19] Alessa M, Almangour TA, Alhassoun A, et al Adherence to evidence-based guidelines for the management of pneumonia in a tertiary teaching hospital in Riyadh. Saudi Pharm J 2023; 31:101678. 37448847 10.1016/j.jsps.2023.06.011 PMC10336669

[20] Arnold FW, LaJoie AS, Brock GN, et al; Community-Acquired Pneumonia Organization (CAPO) Investigators. Improving outcomes in elderly patients with community-acquired pneumonia by adhering to national guidelines: community-acquired pneumonia organization international cohort study results. Arch Intern Med 2009; 169:1515–24. 19752410 10.1001/archinternmed.2009.265

[21] Cilloniz C, Albert RK, Liapikou A, et al The effect of macrolide resistance on the presentation and outcome of patients hospitalized for *Streptococcus pneumoniae* pneumonia. Am J Respir Crit Care Med 2015; 191:1265–72. 25807239 10.1164/rccm.201502-0212OC

[22] Ishiguro T, Takayanagi N, Yamaguchi S, et al Etiology and factors contributing to the severity and mortality of community-acquired pneumonia. Intern Med 2013; 52:317–24. 23370738 10.2169/internalmedicine.52.8830

[23] Jones BE, Ying J, Stevens V, et al Empirical anti-MRSA vs standard antibiotic therapy and risk of 30-day mortality in patients hospitalized for pneumonia. JAMA Intern Med 2020; 180:552–60. 32065604 10.1001/jamainternmed.2019.7495 PMC7042818

[24] Kang SH, Jo YH, Lee JH, Jang DH, Kim YJ, Park I. Antibiotic prescription consistent with guidelines in emergency department is associated with 30-day survival in severe community-acquired pneumonia. BMC Emerg Med 2021; 21:108. 34579649 10.1186/s12873-021-00505-4 PMC8477488

[25] Kobayashi H, Shindo Y, Kobayashi D, et al Extended-spectrum antibiotics for community-acquired pneumonia with a low risk for drug-resistant pathogens. Int J Infect Dis 2022; 124:124–32. 36116670 10.1016/j.ijid.2022.09.015

[26] Lee YT, Chen SC, Chan KC, Wu TC, Tsao SM, Chan CH. Impact of infectious etiology on the outcome of Taiwanese patients hospitalized with community acquired pneumonia. J Infect Dev Ctries 2013; 7:116–24. 23416657 10.3855/jidc.2834

[27] Martin-Loeches I, Lisboa T, Rodriguez A, et al Combination antibiotic therapy with macrolides improves survival in intubated patients with community-acquired pneumonia. Intensive Care Med 2010; 36:612–20. 19953222 10.1007/s00134-009-1730-y

[28] Pflanzner S, Phillips C, Mailman J, Vanstone JR. AMS in the ICU: empiric therapy and adherence to guidelines for pneumonia. BMJ Open Qual 2019; 8:e000554. 10.1136/bmjoq-2018-000554 PMC6542434 31206061

[29] Rello J, Diaz E, Mañez R, et al; CAPUCI II Consortium. Improved survival among ICU-hospitalized patients with community-acquired pneumonia by unidentified organisms: a multicenter case–control study. Eur J Clin Microbiol Infect Dis 2017; 36:123–30. 27655267 10.1007/s10096-016-2779-5

[30] Sims SA, Dale JA, Johnson TJ, Christensen K, Ward E. Electronic quality measurement predicts outcomes in community acquired pneumonia. AMIA Annu Symp Proc 2012; 2012:876–81. 23304362 PMC3540483

[31] Rothberg MB, Zilberberg MD, Pekow PS, et al Association of guideline-based antimicrobial therapy and outcomes in healthcare-associated pneumonia. J Antimicrob Chemother 2015; 70:1573–9. 25558075 10.1093/jac/dku533 PMC4398467

[32] Attridge RT, Frei CR, Restrepo MI, et al Guideline-concordant therapy and outcomes in healthcare-associated pneumonia. Eur Respir J 2011; 38:878–87. 21436359 10.1183/09031936.00141110

[33] Rhee C, Kadri SS, Dekker JP, et al Prevalence of antibiotic-resistant pathogens in culture-proven sepsis and outcomes associated with inadequate and broad-spectrum empiric antibiotic use. JAMA Netw Open 2020; 3:e202899. 32297949 10.1001/jamanetworkopen.2020.2899 PMC7163409

[34] Dellinger RP, Levy MM, Carlet JM, et al Surviving sepsis campaign: international guidelines for management of severe sepsis and septic shock: 2008. Crit Care Med 2008; 36:296–327. 18158437 10.1097/01.CCM.0000298158.12101.41

[35] Haessler S, Lagu T, Lindenauer PK, et al Treatment trends and outcomes in healthcare-associated pneumonia. J Hosp Med 2017; 12:886–91. 29091975 10.12788/jhm.2877 PMC6005651

[36] Jones BE, Jones MM, Huttner B, et al Trends in antibiotic use and nosocomial pathogens in hospitalized veterans with pneumonia at 128 medical centers, 2006–2010. Clin Infect Dis 2015; 61:1403–10. 26223995 10.1093/cid/civ629 PMC4599396

[37] Del Rio-Pertuz G, Gutiérrez JF, Triana AJ, et al Usefulness of sputum Gram stain for etiologic diagnosis in community-acquired pneumonia: a systematic review and meta-analysis. BMC Infect Dis 2019; 19:403. 31077143 10.1186/s12879-019-4048-6 PMC6509769

[38] van der Eerden MM, Vlaspolder F, de Graaff CS, et al Comparison between pathogen directed antibiotic treatment and empirical broad spectrum antibiotic treatment in patients with community acquired pneumonia: a prospective randomised study. Thorax 2005; 60:672–8. 16061709 10.1136/thx.2004.030411 PMC1747487

[39] Peto L, Nadjm B, Horby P, et al The bacterial aetiology of adult community-acquired pneumonia in Asia: a systematic review. Trans R Soc Trop Med Hyg 2014; 108:326–37. 24781376 10.1093/trstmh/tru058 PMC4023908

[40] Arancibia F, Bauer TT, Ewig S, et al Community-acquired pneumonia due to gram-negative bacteria and *Pseudomonas aeruginosa*: incidence, risk, and prognosis. Arch Intern Med 2002; 162:1849–58. 12196083 10.1001/archinte.162.16.1849

[41] Ruiz M, Ewig S, Marcos MA, et al Etiology of community-acquired pneumonia. Am J Respir Crit Care Med 1999; 160:397–405. 10430704 10.1164/ajrccm.160.2.9808045

[42] Cilloniz C, Ewig S, Polverino E, et al Microbial aetiology of community-acquired pneumonia and its relation to severity. Thorax 2011; 66:340–6. 21257985 10.1136/thx.2010.143982

[43] Baquero F . Pneumococcal resistance to β-lactam antibiotics: a global geographic overview. Microb Drug Resist 1995; 1:115–20. 9158743 10.1089/mdr.1995.1.115

[44] Hoban DJ, Doern GV, Fluit AC, Roussel-Delvallez M, Jones RN. Worldwide prevalence of antimicrobial resistance in *Streptococcus pneumoniae*, *Haemophilus influenzae*, and *Moraxella catarrhalis* in the SENTRY antimicrobial surveillance program, 1997–1999. Clin Infect Dis 2001; 32(suppl 2):S81–93. 11320449 10.1086/320181

[45] Kim SH, Song JH, Chung DR, et al Changing trends in antimicrobial resistance and serotypes of *Streptococcus pneumoniae* isolates in Asian countries: an Asian Network for Surveillance of Resistant Pathogens (ANSORP) study. Antimicrob Agents Chemother 2012; 56:1418–26. 22232285 10.1128/AAC.05658-11 PMC3294909

[46] Iorio A, Spencer FA, Falavigna M, et al Use of GRADE for assessment of evidence about prognosis: rating confidence in estimates of event rates in broad categories of patients. BMJ 2015; 350:h870–h870. 25775931 10.1136/bmj.h870

[47] Kern DM, Davis J, Williams SA, et al Validation of an administrative claims-based diagnostic code for pneumonia in a US-based commercially insured COPD population. Int J Chron Obstruct Pulmon Dis 2015; 10:1417–25. 26229461 10.2147/COPD.S83135 PMC4516198

[48] Rodriguez-Barradas MC, McGinnis KA, Akgün K, et al Validation for using electronic health records to identify community acquired pneumonia hospitalization among people with and without HIV. Pneumonia 2020; 12:6. 32724760 10.1186/s41479-020-00068-1 PMC7382068

[49] Drahos J, Vanwormer JJ, Greenlee RT, Landgren O, Koshiol J. Accuracy of *ICD-9-CM* codes in identifying infections of pneumonia and herpes simplex virus in administrative data. Ann Epidemiol 2013; 23:291–3. 23522903 10.1016/j.annepidem.2013.02.005 PMC3654522

